# Swimming suppresses hepatic vitellogenesis in European female silver eels as shown by expression of the estrogen receptor 1, vitellogenin1 and vitellogenin2 in the liver

**DOI:** 10.1186/1477-7827-8-27

**Published:** 2010-03-19

**Authors:** Arjan P Palstra, Denhi Schnabel, Maaike C Nieveen, Herman P Spaink, Guido van den Thillart

**Affiliations:** 1 Molecular Cell Biology, Institute of Biology, Leiden University (IBL), Einsteinweg 55, 2333 CC Leiden, The Netherlands; 2 Departament de Fisiologia, Facultat de Biologia, Universitat de Barcelona, Av. Diagonal 645, 08028 Barcelona, Spain; 3 Departamento Genética del Desarrollo y Fisiología Molecular, Instituto de Biotecnología/UNAM, Av. Universidad #2001, Col. Chamilpa C.P. 62210, Cuernavaca, Morelos, Mexico

## Abstract

**Background:**

When European silver eels (Anguilla anguilla) venture into the Atlantic Ocean for their 6,000 km semelparous spawning run to the Sargasso Sea, they are still in a prepubertal stage. Further sexual development appears to be blocked by dopaminergic inhibition of hypothalamus and pituitary activity. Recently, we found that swimming for several weeks in freshwater stimulated the incorporation of fat droplets in the oocytes. So, it was hypothesized that long term swimming in seawater would release the inhibition further and would also stimulate the production of vitellogenin by the liver.

**Methods:**

For this study a swim-flume was constructed to allow simulated migration of migratory female silver eels for 3 months (1,420 km) in natural seawater at 20 degrees C. Primers were designed for polymerase chain reactions to measure the mRNA expression of estrogen receptor 1 (esr1), vitellogenin1 (vtg1) and vitellogenin2 (vtg2) genes in the liver of European female silver eels.

**Results:**

In comparison to resting eels, swimming eels showed a diminished expression of esr1, vtg1 and vtg2 in the liver. They also had lower plasma calcium (Ca; indicative of vitellogenin) levels in their blood. This showed that vitellogenesis is more strongly suppressed in swimming than in resting eels. However, when eels were subsequently stimulated by 3 weekly carp pituitary extract injections, the expression of the same genes and plasma levels of Ca strongly increased in both groups to similar levels, thus equalizing the initial differences between resting and swimming.

**Conclusions:**

It is concluded that vitellogenesis remains suppressed during resting and even more during swimming. The fact that swimming stimulates fat deposition in the oocytes but suppresses vitellogenesis indicates that these events are separated in nature and occur sequentially. Swimming-suppressed vitellogenesis may imply that in nature eels undergo vitellogenesis and final maturation near or at the spawning grounds.

## Background

When European silver eels (*Anguilla anguilla*) venture into the Atlantic Ocean for their 6,000 km semelparous spawning run to the Sargasso Sea, they are still in a prepubertal stage. When they are prevented from migration, further sexual development e.g. gonad growth and vitellogenesis, appears to be blocked by dopaminergic inhibition of hypothalamus and pituitary activity [1]. Apparently, specific triggers occurring during or after migration are required for further maturation. Dopaminergic inhibition in European eels is early in gametogenesis in comparison with various other teleosts [2] and the induction of full sexual maturation requires 3-6 months of weekly injections with pituitary extracts [3-5]. Treatment with pituitary extracts leads to abnormal oogenesis in Japanese eels as illustrated by variations in yolk accumulation, egg membrane formation and hormone levels [6]. When stimulated with pituitary extract, which contains FSH as well as LH, vitellogenesis is instantly induced as fat droplets are deposited at the same time with yolk globules [6,7]. In nature the two processes usually occur sequentially. So, it is quite possible that the poor success in eel reproduction is related to inadequate activation of vitellogenesis. We recently found evidence that swimming in freshwater (FW) releases (at least partly) reproductive inhibition in female silver eels. Van Ginneken et al. [8] observed increased levels of pituitary LH and plasma 17β-estradiol (E_2_) in 3 year old farmed eels after swimming 5,500 km in freshwater. Older eels (13-21 years from Lake Balaton) were much more sensitive; already after a few weeks of swimming in freshwater, the gonad mass of these silver eels had increased and oocytes had become larger. After two and six weeks of swimming an additional enlargement of the eyes occurred, which is a sign of sexual maturation, and large amounts of fats were incorporated in the oocytes [9]. Vitellogenic yolk globuli did not occur in the oocytes of the swimmers during the first 6 weeks of swimming, while clearly lipid droplets were deposited. These observations suggest that fat droplets and yolk globuli should be deposited sequentially in eel. The first process can be initiated by the fats fueling swimming activity, while the second process may be activated later by maturation hormones. As the last process did not start during the first few weeks of swimming in FW, we assumed that a longer swimming period of 3 months in seawater (SW) might be required to initiate vitellogenesis.

Recently Lokman et al. [10] and Endo et al. [11] showed that 11-ketotestosteron (11-KT) has an important role in lipid transfer and deposition in the growing previtellogenic oocytes. E_2 _did not show such effects *in vitro *[10] but *in vivo *it did [12]. These effects were not only attributable to E_2 _but probably also to gonadotropins [10]. A significant role for 11-KT in lipid transfer and deposition in the oocytes is thus clear but a role for E_2 _cannot be excluded.

The liver plays a crucial role in the production of circulating lipids and vitellogenin. Activation of both production processes has to precede full gonad maturation. Metabolism of lipids in the liver is mainly related to energy metabolism, including lipolysis, transformation of lipids and production of transport proteins. Hepatic synthesis of vitellogenin is generally induced by ovarian estrogens released from the granulosa cells [13], although involvement of other hormones is also required in most teleosts (reviewed by Babin et al. [14]). Estrogen actions are mediated by nuclear estrogen receptors of which the vertebrate genome contains two types: the estrogen receptor 1 (Esr1) and 2 (Esr2). In European eel we have recently shown that the Esr1 plays an important role in the onset of hepatic vitellogenesis [7], the role of the Esr2 at this stage remains unclear [6,15]. The hormone-receptor complex binds in the hepatocyte nucleus at estrogen responsive elements which results in the activation or enhanced transcription of *vitellogenin1 *(*vtg1*) and *vitellogenin2 *(*vtg2*) [14]. The onset of eel vitellogenesis is indicated by the expression of *esr1 *and subsequently *vtg1 *and *vtg2 *[7]. Migrating silver eels are in a pre-pubertal stage, where vitellogenesis is not yet activated. It is thus to be expected that early maturation must be preceded by activation of the liver.

Just very recently [7], we have developed new molecular primers for demonstration of the expression of the *esr1*, *vtg1 *and *vtg2 *in the liver of *Anguilla anguilla*. Sequences of the *vtg2 *and *esr1 *genes were not previously described for this species. Early vitellogenesis appeared as a 3-step process in which 1) *esr1 *expression was significantly increased already after one injection carp pituitary extract (CPE), 2) *vtg1 *and *vtg2*-expression were significantly increased after one and two injections respectively, and 3) *vtg1 *and *vtg2*-expression increased further after three and four injections when also plasma calcium (corresponds with plasma vitellogenin) increased and yolk globuli appeared in the oocytes.

In this study we have used these molecular primers to investigate the effect of resting and swimming on hepatic vitellogenesis. For this purpose, large migratory female silver eels were subjected to 3 months group-wise swimming in natural seawater in a swim-flume to find time-points of increased hepatic vitellogenic sensitivity. It is hypothesized that swimming should release dopaminergic inhibition in silver eels which should start with activation of hepatic vitellogenesis and up-regulated expression of *esr1*, *vtg1 *and *vtg2*.

## Methods

### Design and construction of a swim-flume

An oval shaped swim-flume (6.0 × 4.0 × 0.8 m) was constructed in a climatized room of 100 m^2 ^to allow group-wise simulated migration. The compartment for swimming eels was placed in the WSW-direction of the Sargasso Sea since eels have been suggested to use the earth magnetic field for orientation and navigation [16]. The total water content of about 6,000 L was recirculated continuously over a bio-filter. Natural seawater was transported from the Oosterschelde at the Burgersluis (the Netherlands) by truck (Van Vugt, Zuilinchem, the Netherlands). A stable and linear water flow profile was created with two SPECK pumps (Type BADU 40/13, Zevenaar, the Netherlands) with a capacity of 13 m^3 ^h^-1^. Stream profiles were checked with a 3D water velocity sensor Vectrino (Qmetrix/NORTEK, Hoofddorp, the Netherlands) by coherent Doppler processing. The illumination in the climatized room was switched to 670 nm light (bandwidth 20 nm) as described by van den Thillart et al. [17]. Based on eye pigment changes during silvering, this far-red light is invisible for eels [18].

The water temperature was set at 20°C. Salinity of the water during the experimental period was kept at 34.9 ± 1.4 ppt. Water quality was maintained with at pH = 8.2 ± 0.1, NO_3_^- ^= 12.5 mg L^-1^, NO_2_^- ^= 0.1 ± 0.2 mg L^-1^, and NH_4_^+ ^= 0 mg L^-1^.

### Experimental eels and swimming

Experiments complied with the current laws of the Netherlands and were approved by the Dutch animal experimental board (DEC nr. 06059). Migratory female silver eels (n = 54; body-length = 75 ± 1 cm, body-weight = 749 ± 25 g, eye index = 14.3 ± 0.2) from Greece were purchased from a Dutch eel import company (Kraan BV, Leimuiden, The Netherlands) in February 2006. Eels were anaesthetized with 1.5 ml diluted clove oil per L water (oil of cloves: ethanol = 1:10) and subcutaneously injected with PIT-TAGS (TROVAN, Aalten, The Netherlands).

Eels were randomly divided over 9 groups of n = 6 each. One group (control) was measured and sampled at the start. Before the experiment all other eels were treated with an antibiotic (Flumequin; Flumix, Eurovet, Bladel, The Netherlands, 50 mg l^-1 ^for 1-2 h).

Four groups were swum at an average speed of 0.20 m s^-1^, corresponding to 0.27 body-lengths per second (BL s^-1^); two groups swam for 1.5 months and two other groups swam for 3 months. Eels were daily checked for their swimming performance, no problems with swimming were observed. Four other groups were kept resting under the same conditions as the swimmers except for stream in a separate 1,700 L tank connected to a 6,000 L recirculation system with bio-filter, two groups for 1.5 months and two for 3 months. After each period one swim and one rest group were sacrificed, while one swim and one rest group were exposed to a maturation sensitivity test. This test consisted of 3 weekly injections with 20 mg carp pituitary extract (CPE; Catvis, 's-Hertogenbosch, The Netherlands) while resting. This test was performed to compare the experimental groups in a stimulated condition (also Durif et al. [19]), therefore no resting controls without CPE treatment were included. Before each CPE injection, eels were treated with Flumequin as described above. After 3 weeks the CPE treated eels were dissected to determine the effect of CPE treatment on maturation.

### Sampling and dissection

All eels were measured before and after each period for biometric parameters: body-length (BL), body-weight (BW), eye diameter horizontal (EDh), eye diameter vertical (EDv), pectoral fin length (PFL) and pectoral fin width (PFW). From these data the following indices were calculated: 1) Condition factor (K) = 100 × BW BL^-3^, with BW: body weight (g), BL: body length (cm); 2) Eye index (EI) = 100 × (((EDh + EDv) × 0.25)^2 ^π × (10 × BL)^-1^), with EDh: eye diameter horizontal (mm), EDv: eye diameter vertical (mm) [20]; 3) Pectoral fin length index (PFLI) = 100 × PFL BL^-1^, with PFL: pectoral fin length (cm); 4) Pectoral fin width index (PFWI) = 100 × PFW BL^-1^, with PFW: pectoral fin width (cm); 5) The silver index (SI) was calculated according to Durif et al [21].

All eels were sampled for blood before and after each period. Blood samples were taken from the caudal vein with heparin flushed (10.000 IU ml^-1^) 1 ml syringes, which were immediately placed on ice. Blood was centrifuged for 5 min at 14,000 rpm and plasma was stored at -80°C. After treatment eels were sacrificed, liver, digestive tract and gonads were carefully dissected and weighted. Liver tissue (~200 mg) was stored in 1 ml RNA *later *(Ambion, Cambridgeshire, UK) at -20°C for later analysis. From the dissected tissues the following indices were calculated: 1) Gonadosomatic index (GSI) = (GW × BW^-1^) × 100%, with GW: gonad weight (g), BW: body weight (g); 2) Digestive tract somatic index (DTSI) = (DTW × BW^-1^) × 100%, with DTW: weight digestive tract (g); 3) Hepatosomatic index (HSI) = (LW × BW^-1^) × 100%, with LW: liver weight (g).

### Blood plasma Calcium (Ca)

Blood plasma Vtg was measured indirectly through Ca (human kit 10011, Human Gesellschaft fur Biochemica und Diagnostica mbH, Wiesbaden, Germany). A significant positive correlation between Ca and Vtg has been demonstrated for rainbow trout by Verslycke et al. [22] and used on eels by Versonnen et al. [23].

### RNA isolation

Total RNA was isolated from the liver of all RNA *later *samples and extracted using TRIZOL (Invitrogen) reagent. Traces of DNA were removed by incubation with DNAse-I (Ambion). The DNAse treated RNA was transcribed to cDNA according to the description provided by the manufacturer (HT Biotechnology LTD).

### Primers

Primers were designed from the recently cloned partial sequences for European eel of *esr1 *(Genbank EU073125), of *vtg1 *(Genbank EU073127) and of *vtg2 *(Genbank EU073128). The primer sequences were recently published by Palstra and Schnabel et al. [7]. The primer for *β actin *was designed from the reported sequence of *Anguilla japonica *(Kurokawa; Genbank AB074846).

### Quantitative RT-PCR

Quantitative RT-PCR was performed using the qPCR MasterMix for SYBR^® ^Green I (Eurogenetec). The conditions in the Chromo 4™ Detector (Bio-Rad laboratories) used were 95°C during 10 min followed by 40 cycles of denaturation at 95°C for 15 s, annealing temperature at 55°C for 30 s and extension at 72°C for 40 s and the reading of the plate in each cycle, followed by a final step at 65°C for 10 min. To show that only one final product was amplified, a melting curve from 65°C to 95°C holding during 5 s each 0.5°C, was generated. The data were analyzed using the opticon monitor 3 program (Bio-Rad laboratories).

Standard curves were generated from the plasmids containing each of the specific genes *β actin*, *esr1*, *vtg1 *and *vtg2 *for each well plate for the Ct values vs. the logarithmic total DNA. R^2^-values were for all standard curves >0.98. The equation was used to calculate the corresponding amount of DNA for the mean of each duplicate of each sample. The supposed housekeeping gene *β actin *was checked for its functioning as a housekeeper. No significant differences existed in Ct values of *β actin *between the different groups that were compared (average Ct ± standard error 16.85 ± 0.08). DNA amounts could therefore be normalized to the expression level of the housekeeping gene *β actin *(between x and 100%). All samples were measured in duplicate for each of the genes of choice. Per gene, three well-plates with samples were subjected to quantitative RT-PCR, each containing a standard curve plus 1) control group, 2) swim and rest groups, and 3) swim and rest plus CPE injections - groups. Data were expressed as fold change of swim-groups vs. rest-groups to exclude the effects of time.

### Analysis and statistics

Data were checked by Kolmogorov-Smirnov tests for normality. Changes in morphometry and Ca were analysed before and after each treatment (e.g. swimming, CPE injections). Since all occurred in the same individual eels, data were statistically compared using paired one-tailed t-tests. Changes of internal parameters and expression of target genes were compared between different individual eels and analysed using unpaired tests. Changes of internal parameters (HSI, DTSI and GSI) were statistically compared using Mann-Whitney U-tests with one-tailed probabilities. Non-responders to CPE injections were identified by the absence of an increase in GSI. With these tests, their pooled values for maturation parameters were compared with the pooled values of the responding eels that received CPE injections. For analysis of gene expression in CPE-treated eels, data of non-responders were excluded from analysis. No significant correlations were found between size and gene expression (quantitative RT-PCR) so unpaired one-tailed t-tests were performed instead of ANCOVA. All statistical tests were performed in SPSS 12.0 for Windows. All results were expressed as average ± standard error (av ± se). P-values ≤ 0.05 were considered to indicate statistically significant differences.

## Results

### Swimming behaviour and distance

**Figure 1:**
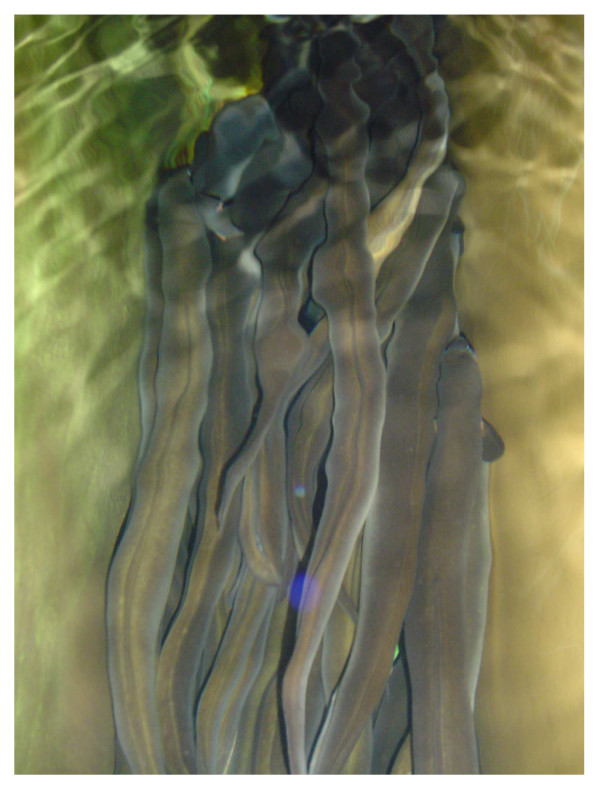
**Group-wise simulated migration of large female silver eels**. Twenty-four large female silver eels swimming in natural seawater for 907 km (n = 12) and 1,420 km (n = 12) in the newly constructed 6,000 L swim-flume.

The newly constructed swim-flume proved to be very useful for subjecting large groups of eels to long term simulated migration (Figure 1). During the three months of simulated migration, none of the eels stopped swimming. At each daily check all eels were swimming, except in some incidental cases when a single eel was resting temporarily against the back screen. Such eels started to swim immediately when stimulated by flashlight. Eels schooled and swam in a dense group near the front screen in the lower half of the water layer. Eels swam for a certain period in the front line after which they rose in the water layer, slowed down speed, joined up the last line and were replaced by the next line of eels. After 1.5 months (47 days), the swimmers had covered a distance of 907 km. After 3 months (94 days), the remaining eels had covered a distance of 1,420 km. No eels dropped out during the swim trials. No mortality occurred in the rest or in the swim group.

### Effect of resting

Eels that rested for 1.5 months had a significantly decreased BW (-4.9%) and K (-4.7%; Table 1) in comparison to the control (start conditions) group. For eels that had rested for 3 months, these changes were nearly doubled (BW: -8.4%, K: -8.6%). The eye index of the rested eels was significantly lower (-6.1%) after three months. No significant changes were observed in Ca levels of resting eels (Table 1). All rest groups had significantly lower HSIs and GSIs compared to the control group (Table 2).

**Table 1:** Biometric and blood parameters

a)		start	1.5-mnths rest	1.5-mnths rest and CPE
	BL (cm)	76 ± 2	76 ± 2	76 ± 2
	BW (g)	753 ± 44	**716 ± 43**	715 ± 45
	K	0.169 ± 0.006	**0.161 ± 0.006**	0.160 ± 0.006
	EI	15.2 ± 0.5	15.0 ± 0.5	15.4 ± 0.4
	PFLI	5.53 ± 0.17	5.49 ± 0.15	5.45 ± 0.15
	PFWI	2.99 ± 0.10	2.98 ± 0.13	3.06 ± 0.16
	Is	5	5	5
	Ca (mM)	2.45 ± 0.03	2.40 ± 0.07	**3.45 ± 0.03**
b)		start	1.5-mnths swim	1.5 mnths swim and CPE
	BL (cm)	77 ± 2	77 ± 2	77 ± 2
	BW (g)	770 ± 87	**733 ± 76**	**744 ± 81**
	K	0.169 ± 0.005	**0.161 ± 0.005**	**0.163 ± 0.006**
	EI	14.1 ± 1.1	14.6 ± 1.5	14.8 ± 1.3
	PFLI	5.41 ± 0.04	5.42 ± 0.09	5.36 ± 0.12
	PFWI	2.82 ± 0.14	2.85 ± 0.08	2.83 ± 0.10
	Is	5	5	5
	Ca (mM)	2.51 ± 0.08	2.36 ± 0.03	**2.83 ± 0.20**
c)		start	3-mnths rest	3 mnths rest and CPE
	BL (cm)	75 ± 2	75 ± 1	75 ± 2
	BW (g)	678 ± 36	**621 ± 16**	623 ± 35
	K	0.163 ± 0.008	**0.149 ± 0.004**	0.149 ± 0.007
	EI	14.7 ± 0.7	**13.8 ± 0.3**	**15.0 ± 0.4**
	PFLI	5.32 ± 0.05	5.37 ± 0.02	5.11 ± 0.27
	PFWI	2.85 ± 0.03	2.76 ± 0.02	2.76 ± 0.06
	Is	5	5	5
	Ca (mM)	2.46 ± 0.07	2.42 ± 0.06	2.79 ± 0.20
d)		start	3 mnths swim	3 mnths swim and CPE
	BL (cm)	76 ± 3	76 ± 1	76 ± 3
	BW (g)	849 ± 134	**802 ± 60**	790 ± 137
	K	0.189 ± 0.010	**0.178 ± 0.005**	0.175 ± 0.012
	EI	15.5 ± 0.7	14.9 ± 0.3	15.6 ± 0.6
	PFLI	5.54 ± 0.14	**5.47 ± 0.06**	5.54 ± 0.17
	PFWI	2.75 ± 0.13	2.71 ± 0.06	2.81 ± 0.09
	Is	4.60 ± 0.24	4.60 ± 0.11	4.60 ± 0.24
	Ca (mM)	2.56 ± 0.11	**2.35 ± 0.06**	**2.80 ± 0.25**

a) at the start, after 1.5 months resting and after 3 subsequent CPE injections in the same eels, b) at the start, after 1.5 months swimming and after 3 subsequent CPE injections in the same eels, c) at the start, after 3 months resting and after 3 subsequent CPE injections in the same eels and d) at the start, after 3 months swimming and after 3 subsequent CPE injections in the same eels. Comparisons were made in subsequent order; rest vs. start and rest + CPE vs. rest, as paired data. Statistical significant differences at P ≤ 0.05 are given in bold.

(BL = body-length, BW = body-weight, K = condition factor, EI = eye index, PFLI = pectoral fin length index, PFWI = pectoral fin width index, Is = silver index, Ca = calcium).

**Table 2:** HSI, DTSI and GSI

a)		start	
	HSI	0.89 ± 0.03	
	DTSI	1.03 ± 0.12	
	GSI	1.47 ± 0.08	
b)		1.5 mnths rest	1.5 mnths rest + CPE
	HSI	0.75 ± 0.04^ab^	0.98 ± 0.03^ab^
	DTSI	1.11 ± 0.13^b^	0.73 ± 0.04^ab^
	GSI	1.08 ± 0.09^abc^	2.24 ± 0.28^ab^
c)		1.5 mnths swim	1.5 mnths swim + CPE
	HSI	0.87 ± 0.07	1.02 ± 0.05^a^
	DTSI	0.97 ± 0.07^b^	0.74 ± 0.05^ab^
	GSI	1.34 ± 0.06^bc^	2.25 ± 0.28^ab^
d)		3 mnths rest	3 mnths rest + CPE
	HSI	0.74 ± 0.02^a^	0.89 ± 0.03^b^
	DTSI	1.20 ± 0.11^bc^	0.66 ± 0.09^ab^
	GSI	1.11 ± 0.08^ab^	1.97 ± 0.16^ab^
e)		3 mnths swim	3 mnths swim + CPE
	HSI	0.72 ± 0.04^b^	0.87 ± 0.04^b^
	DTSI	0.93 ± 0.02^c^	0.74 ± 0.09^a^
	GSI	1.13 ± 0.06^ab^	1.94 ± 0.29^b^

a) eels dissected at the start, b) eels dissected after 1.5 months resting and eels dissected after 3 subsequent CPE injections, c) eels dissected after 1.5 months swimming and eels dissected after 3 subsequent CPE injections, d) eels dissected after 3 months resting and eels dissected after 3 subsequent CPE injections, e) eels dissected after 3 months swimming and eels dissected after 3 subsequent CPE injections. The rest or swim groups and the groups after subsequent CPE injections were compared with the start values (statistical differences at P ≤ 0.05 indicated by a) and between them (statistical differences indicated by b). Swim groups were also compared with rest groups (statistical differences indicated by letter c). HSI = hepatosomatic index, DTSI = digestive tract somatic index, GSI = gonadosomatic index.

In comparison to the controls, the gene expression for *esr1 *was higher in the 1.5 months rest group but showed high individual variation in the 3 months rest group (Table 3). *Vtg1 *expression remained similar over time in resting eels. Expression of *vtg2 *was slightly but significantly increased in both rest groups in comparison to the control group.

**Table 3:** Gene expression during resting

	*esr1 *(× 10^-5 ^ng)		*vtg1 *(× 10 ng)		*vtg2 *(× 10^-2 ^ng)	
	av	se	av	se	av	se
control	1.53	0.13	1.46	0.12	1.27	0.10
1.5 mnths rest	**2.50**	**0.39**	1.52	0.45	**2.90**	**0.49**
3 mnths rest	7.76	3.69	1.66	0.43	**3.07**	**0.32**

Expression of *esr1*, *vtg1 *and *vtg2 *in the controls, after 1.5 months resting and after 3 months resting. Statistical significant differences at P ≤ 0.05 of resting eels vs. controls are given in bold.

### Effect of swimming

After 1.5 months swimming, eels showed a significantly lower BW (-3.4%) and K (-4.7%; Table 1). For eels that had swum for 3 months the decreases in BW and K were only slightly higher (BW: -5.5%, K: -5.8%). Ca levels were significantly lower after swimming for 3 months (-8.2%; Table 1).

Eels that swam for 1.5 months had a significantly higher GSI than resting eels (1.34 ± 0.14 vs. 1.08 ± 0.09; Table 2). Eels that swam for 3 months did not show a higher GSI but had a significantly lower DTSI than resting eels (0.93 ± 0.04 vs. 1.20 ± 0.27).

**Figure 2:**
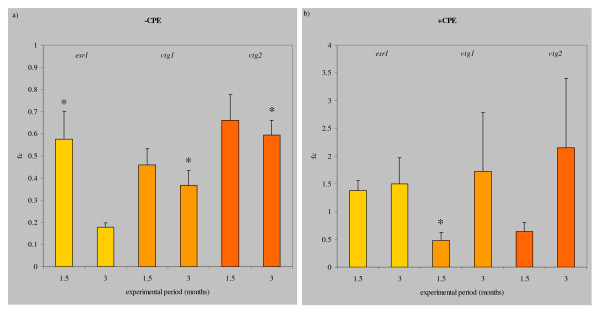
**Effects of swimming exercise on the expression of *estrogen receptor 1*, *vitellogenin1 *and *vitellogenin2***. Relative expression of *estrogen receptor 1*, *vitellogenin1 *and *vitellogenin2 *(fold change fc of swimmers vs. resters) after a) swimming for 1.5 and 3 months (-CPE), and b) after subsequent stimulation with three weekly CPE injections (+CPE). Asterisks indicate a significant difference (P < 0.05) in ng mRNA of a swim group vs. the rest group. Expression of all genes is reduced after swimming 1.5 months and more after swimming 3 months vs. expression in the resting eels (fc<1). CPE treatment compensates for these differences equalizing the initial differences between resting and swimming (fc>1).

Expression of *esr1*, *vtg1 *and *vtg2 *were significantly lower in swim groups in comparison with the rest groups (Figure 2). Expression patterns of both forms of *vtg *were similar.

### Effect of swimming and resting on maturation sensitivity

No mortality occurred during the three weeks of CPE stimulation. After CPE injections, the EI had increased significantly in the eels that rested for 3 months (Table 1). BW and K remained similar, except for eels that swam for 1.5 months. They showed a slightly increased BW and K after injections (Table 1). Ca levels were generally higher after injections (Table 1). The reaction was strongest after 1.5 months of resting (+36%) and swimming (+16%; Table 1). After 3 months, eels showed 15.3% increase after resting (ns) and 19.1% after swimming.

GSIs were significantly higher after injections for both groups (Table 2). After 1.5 months resting plus injections, the GSI was 2.24 ± 0.28 vs. 1.08 ± 0.09 of controls and after 1.5 months swimming plus injections 2.25 ± 0.28 vs. 1.34 ± 0.06. After 3 months resting plus injections, the GSI was 1.97 ± 0.16 and after 3 months swimming plus injections 1.94 ± 0.29. No significant differences were apparent between the resting and swimming eels after injections in GSI, and also not in HSI and DTSI.

In each of the four experimental groups that received CPE injections, one individual did not respond; in total four individuals. These non-responders did not show a change in any of the maturation parameters typical for CPE treatment, changes that were significant in all other responding eels with lower DTSI (P = 0.01) and higher GSI (P < 0.001) and plasma Ca (P < 0.001). So, data of the few non-responders could be left out from statistical analysis.

After the CPE injections, expression of the three genes was over 5-fold higher in all groups (P < 0.01) in comparison with the non CPE-treated groups. Now expression in swimmers was relatively stronger for all target genes: *esr1 *was higher after 1.5 months and after 3 months while *vtg1 *and *vtg2 *expression were still lower after 1.5 months swimming but higher after 3 months swimming as compared to resting eels. Individual variation among 3 months swimmers was however high: for expression of each of the target genes, three or four eels that swam fell in the range of the resters but one (*vtg1*) or two individuals (*esr1*, *vtg2*) scored much higher.

## Discussion

In recent experiments with silver eels from Lake Balaton, we observed after 6 weeks of swimming in fresh water (FW) a significant increase of GSI and HSI, and a decrease of DTSI in comparison with resting eels [9]. In that study we found that swimming stimulated the incorporation of large amounts of fats. However, it did not stimulate vitellogenesis since plasma Vtg levels remained low [24] and yolk globuli were not present in the oocytes. Also the oocyte diameters remained smaller than 250 μm, which is considered a requirement for the onset of vitellogenesis [6]. Therefore, we assumed that continued swimming, particularly in combination with the transfer to seawater, would induce vitellogenesis. Transfer to sea water is used to activate silvering of farmed eels before starting artificial maturation [25]. As vitellogenesis has to start in the liver with the production of vitellogenin, we developed specific primers to follow this process in its early phase [7]. Hepatic vitellogenesis requires the expression of *esr1*, *vtg1 *and *vtg2 *genes. Furthermore, vitellogenin release to the blood stream should become visible by increased Ca levels in the plasma.

Resting eels in this study had after 3 months a lower EI, a lower HSI and a lower GSI (Table 1 and 2). This clearly shows regression of the silver status. In nature silver eels that do not leave in autumn show regression of their silver status when the water temperature starts to rise in spring. These eels start feeding again, while maturation parameters decrease and the DTSI (digestive tract somatic index) increases [21,26]. Experimental eels in this study were caught at the end of February at low water temperatures. However, our experimental water temperature was 20°C, which may have contributed to a faster regression.

The results of this study show that hepatic vitellogenesis was not stimulated by swimming in seawater for 1.5 and 3 months. Moreover, hepatic vitellogenesis was even reduced after swimming. The expression of *esr1 *was lower in swimming eels vs. resting eels. This reduction in expression in swimmers may have caused the reduced expression of *vtg1 *and *vtg2*. Also from the resulting blood plasma Ca levels it can be concluded that swimming suppresses vitellogenesis. Since swimming did not stimulate hepatic vitellogenesis, our hypothesis has to be rejected. We can conclude that seawater (SW)-swimming even suppresses vitellogenesis and that dopaminergic inhibition should still be active in female silver eels after 3 months SW swimming.

Supporting arguments come from a parallel study [27] in which we investigated the LHβ and FSHβ expression in the pituitaries of females and their oocyte histology. Dopaminergic inhibition of pituitary activity was still effective since LHβ and FSHβ expression in the pituitaries was similar for the resting and swimming females. The same accounted for oocyte diameters. The absence of swimming-induced oocyte development is in line with the reduced *esr1*, *vtg1 *and *vtg2 *expression in this study. As a result, Ca levels decreased and yolk was not incorporated in the oocytes.

The mechanism behind swimming-suppressed vitellogenesis may be explained through the action of cortisol. Cortisol is well-known for its action in lipid mobilisation which is necessary to fuel swimming and for the swimming-induced fat deposition in the oocytes which was found for silvering eels in fresh water (FW) [9]. These eels indeed showed elevated cortisol levels after six weeks swimming [24]. Swimming may have up-regulated mammalian GnRH through cortisol, binding to the glucocorticoid receptor [28,29]. Seawater swimming did however not lead to increased LHβ or FSHβ expression in the pituitary of females [27], probably since the female pituitary was still under dopaminergic control. Without increase of LH production and release, the ovaria do not mature further, plasma E_2 _remains low and the liver does not become sensitive. Cortisol could also have direct effects on vitellogenesis since it was found to inhibit E_2_-induced vitellogenin synthesis in the rainbow trout, an effect mediated by a decrease in *esr1 *(previously known as the *estradiol receptor α*) mRNA levels [30]. The role of cortisol in swimming-suppressed vitellogenesis should be clarified in future experiments.

In eels, it may well be that E_2 _alone is not enough for inducing vitellogenesis. Van Ginneken et al. [8] found that FW swimming of farmed eels leads to increased E_2 _levels but Vtg levels remained low. Also administration of solely estrogens to silver eels has never succeeded in induction of progression of gonadal development as resulting from vitellogenesis [12,31-33]. *In vitro *experiments on immature eel hepatocytes revealed that E_2_-induction depended on presence of growth hormone (GH) or prolactin [14,34,35]. Also FSH might be involved in triggering eel vitellogenesis as suggested by Kamei et al. [36,37], for instance by regulating *esr1 *expression. For the female eels in this study we found that SW swimming did not enhance FSHβ expression in the pituitary [27]. The FSH-lack may prevent eels from starting vitellogenesis independent of E_2 _levels in the blood. Supporting evidence comes from a recent experiment where we have investigated the effect of 4 weekly injections of Human Chorionic Gonadotropin (hCG; functional analogue to LH) on the vitellogenic response. This treatment increased the E_2 _levels in the blood over 40 times but no yolk globuli were found in the oocytes (Palstra et al., unpublished results). This may also indicate that increased E_2 _levels are not enough for Vtg production. Clearly the estrogen receptors were not expressed, suggesting that a stimulus by FSH is required. FSHβ expression in European eels has been shown to correlate negatively to water temperature [38]. Future SW swimming trials should therefore be performed at lower temperatures.

When swimming and resting eels were stimulated after 3 months by CPE injections, the expression of all tested genes and plasma Ca strongly increased. The LH and FSH from CPE apparently circumvents the female eel pituitary that likely remains under dopaminergic inhibition. Independently from the initial degree of maturation, CPE initiates vitellogenesis rapidly (also [7]). This may explain why CPE-treatment does not reveal a difference between swimming and resting. On the other hand, preliminary results (Palstra et al., unpublished results) showed that after CPE-injections up to full maturation, swimmers ovulated on average 2-3 weeks later than the resters.

It may thus well be that during oceanic migration, vitellogenesis is blocked. During endurance swimming lipid mobilisation is activated, which allows the transport to and the deposition of lipids in the oocytes. The production of Vtg during this process might compete for the same source resulting in too small lipid content in the oocytes. High fat stores in the oocyte will likely improve the gamete quality and survival chances for the early larvae. On the other hand, side effects of vitellogenesis may impair swimming capacity due to: 1) bone resorption [39], 2) muscle atrophy [40,41], and 3) enlargement of the belly. The latter would increase the drag and therefore the cost of transport. Swimming-suppressed vitellogenesis would imply that in nature eels undergo vitellogenesis and final maturation near or at the spawning grounds, most possibly further stimulated by the presence of maturing males and the high temperature of the Sargasso Sea.

Eels migrating in the ocean display a diel vertical migration that has been shown repeatedly during tracking experiments using pop-up tag methodology [42-45]. Eels were escaping light by ascending during dusk and descending during dawn at depths between 200 and 700 m, both in continental and deep-sea waters which probably persists as far as the spawning grounds. So, besides the effects of swimming as discussed in this study, the diel vertical migration in the ocean can be expected to have effects on sexual maturation through the alternation in pressure [46], temperature (Tsukamoto K, presented at the Aquaculture Europe 2008 symposium organised by the European Aquaculture Society in Krakow, Poland; [38]) and avoidance of light [47].

## Conclusions

Swimming in seawater suppresses hepatic vitellogenesis in female silver eels and dopaminergic inhibition should still be active after 3 months swimming. Hormonal stimulation after exercise provoked a rapid catch-up of hepatic vitellogenesis and reproductive development in general. The fact that swimming stimulates fat deposition in the oocytes but suppresses vitellogenesis indicates that these events are separated in nature and occur sequentially. Sexual maturation of female silver eels may therefore progress near or at the spawning site.

## Competing interests

The authors declare that they have no competing interests.

## Authors' contributions

APP and GVDT conceived and designed the project and the experiments. APP performed the experiments, measurements and dissection. DS and HPS tested the right conditions for Quantitative RT-PCR. APP and MCN performed Quantitative RT-PCR. APP and GVDT wrote the paper. All authors read and approved the final manuscript.
